# Simultaneous fermentation of cellulose and current production with an enriched mixed culture of thermophilic bacteria in a microbial electrolysis cell

**DOI:** 10.1111/1751-7915.12733

**Published:** 2017-05-29

**Authors:** Bradley G. Lusk, Alexandra Colin, Prathap Parameswaran, Bruce E. Rittmann, Cesar I. Torres

**Affiliations:** ^1^ Biodesign Swette Center for Environmental Biotechnology Arizona State University P.O. Box 875701 Tempe AZ 85287‐5701 USA; ^2^ #ScienceTheEarth Mesa AZ 85201 USA; ^3^ Ecole Normale Superieure 45, rue d'Ulm 75230 Paris Cedex 05 France; ^4^ Department of Civil Engineering Kansas State University 2123 Fiedler Hall Manhattan KS 66502 USA; ^5^ School of Sustainable Engineering and the Built Environment Arizona State University Tempe AZ USA; ^6^ School for Engineering of Matter, Transport and Energy Arizona State University Tempe AZ USA

## Abstract

An enriched mixed culture of thermophilic (60°C) bacteria was assembled for the purpose of using cellulose to produce current in thermophilic microbial electrolysis cells (MECs). Cellulose was fermented into sugars and acids before being consumed by anode‐respiring bacteria (ARB) for current production. Current densities (*j*) were sustained at 6.5 ± 0.2 A m^−2^ in duplicate reactors with a coulombic efficiency (CE) of 84 ± 0.3%, a coulombic recovery (CR) of 54 ± 11% and without production of CH_4_. Low‐scan rate cyclic voltammetry (LSCV) revealed a mid‐point potential (*E*_*ka*_) of −0.17 V versus SHE. Pyrosequencing analysis of the V4 hypervariable region of 16S rDNA and scanning electron microscopy present an enriched thermophilic microbial community consisting mainly of the phylum Firmicutes with the *Thermoanaerobacter* (46 ± 13%) and *Thermincola* (28 ± 14%) genera occupying the biofilm anode in high relative abundance and *Tepidmicrobium* (38 ± 6%) and *Moorella* (11 ± 8%) genera present in high relative abundance in the bulk medium. The *Thermoanaerobacter* (15 ± 16%) and *Brevibacillus* (21 ± 30%) genera were also present in the bulk medium; however, their relative abundance varied by reactor. This study indicates that thermophilic consortia can obtain high CE and CR, while sustaining high current densities from cellulose in MECs.

## Introduction

Plant biomass, the most abundant biopolymer on Earth, consists of 3–30% lignin, 30–56% cellulose and 10–27% hemicellulose (Emtiazi and Nahvi, 2000; Niessen *et al*., 2005; Carere *et al*., 2008). Harnessing energy from plant biomass is difficult, as the glycan polymers of which it is composed are difficult to biodegrade (Olson *et al.,* 2012; Basen *et al*., 2014). Cellulose, for example, is only susceptible to degradation from organisms containing cellulolytic enzymes or cellulases (Niessen *et al*., 2005; Carere *et al*., 2008). For this reason, many conventional methods for extracting energy from plant biomass consist of combustion processes that produce large amounts of ash and are highly regulated (Badger, 2002). In contrast, bioprocessing uses non‐combustion biotechnologies to harness the energy stored in plant biomass to produce beneficial fermentation products, including electricity, CH_4_, ethanol, acetate or hydrogen gas (Demain *et al*., 2005; Wilson, 2009; Li *et al*., 2012; Xia *et al*., 2012; Hama *et al*., 2014; Saripan and Reungsang, 2014).

Consolidated bioprocessing is a rapidly advancing field that uses bacteria to produce ethanol in high concentrations on an industrial scale from cellulosic biomass in one step without exogenous cellulase enzymes (Olson *et al.,* 2012). Another employed biological technology utilizes isolated cellulolytic enzymes to degrade cellulose into glucose that is then fermented by yeast to produce high ethanol concentrations; however, the process of purifying these enzymes is costly (Olson *et al.,* 2012). Thermophilic bacterial consortia have been identified as ideal candidates for consolidated bioprocessing as they can degrade cellulose at higher activities than isolated enzymes (Bryant, 2011; Zambare *et al*., 2011) and can operate at slightly acidic, neutral or slightly basic pH conditions (Lynd *et al*., 2002; Sizova *et al*., 2011; Lusk *et al*., 2016).

Microbial electrochemical cells (MxCs) utilize anode‐respiring bacteria (ARB) that are capable of consuming sugars, acids and alcohols for the production of electrical current (*j*) via anode respiration (Oh and Logan, 2005; Kim *et al*., 2007; Mathis *et al*., 2008; Parameswaran *et al*., 2013; Lusk *et al*., 2015, 2016). Previous studies have reported that the products of cellulose fermentation can be utilized in MxCs for the generation of current or hydrogen gas using mixed cultures (Niessen *et al*., 2005; Rismani‐Yazdi *et al*., 2007; Ren *et al*., 2008). Coupling ARB with cellulolytic bacteria provides the possibility of converting cellulose directly into current in an MxC without having to collect fermentation products. In addition, MxCs may decrease inhibition caused by the accumulation of acids from cellulose fermentation (Demain *et al*., 2005; Niessen *et al*., 2005), as ARB consume these acids to produce current. Previous studies with mesophilic cellulolytic cultures coupled with ARB in MxCs produced low current density – 0.05 A m^−2^ (Ren *et al*., 2008), < 0.18 A m^−2^ (Rismani‐Yazdi *et al*., 2007) and 1.8 A m^−2^ (Niessen *et al*., 2005) – and low or unreported coulombic efficiency (CE). However, several thermophilic bacteria exhibit cellulolytic activity in pure and mixed culture studies (Lynd *et al*., 2002; Demain *et al*., 2005; Kato *et al*., 2005; Sizova *et al*., 2011); thus, using them in thermophilic MxCs may provide enhanced electron recovery and capture efficiency.

As no bacterium is known to be capable of cellulose fermentation and anode respiration, we chose to employ a thermophilic microbial consortium for the efficient conversion of cellulosic material into current. Thermophilic MxCs have the potential to convert cellulose into current with high coulombic recovery (CR) and high CE due to the superior growth kinetics, cellulase activity, stability and diffusion rates of H^+^ within the biofilm anode with thermophilic temperature (McBee, 1950; Mathis *et al*., 2008; Torres *et al*., 2008; Taylor *et al*., 2009; Sizova *et al*., 2011; Parameswaran *et al*., 2013; Lusk *et al*., 2015, 2016). For example, *Thermincola ferriacetica*, a thermophilic ARB capable of producing current from the consumption of acetate, has a doubling time five times faster than *Geobacter sulfurreducens* – a model mesophilic ARB (Parameswaran *et al*., 2013) – has a large pH range (5.2–8.3) (Lusk *et al*., 2016) for growth and can achieve a high *j* (> 8 A m^−2^) (Parameswaran *et al*., 2013; Lusk *et al*., 2016) and CE (93%) in MxCs (Parameswaran *et al*., 2013). In addition, *Thermoanaerobacter pseudethanolicus* is capable of fermenting cellulose degradation products, including glucose and cellobiose, into acetate before ultimately producing current (Lusk *et al*., 2015). This study employed a consortium containing *T. ferriacetica* and *T. pseudethanolicus* with an enriched culture of cellulolytic bacteria for the purpose of showing that higher current densities from cellulose are possible in thermophilic MxCs.

## Results and discussion

### Initial growth and current production from cellulose‐fed microbial electrolysis cells

**Figure 1:**
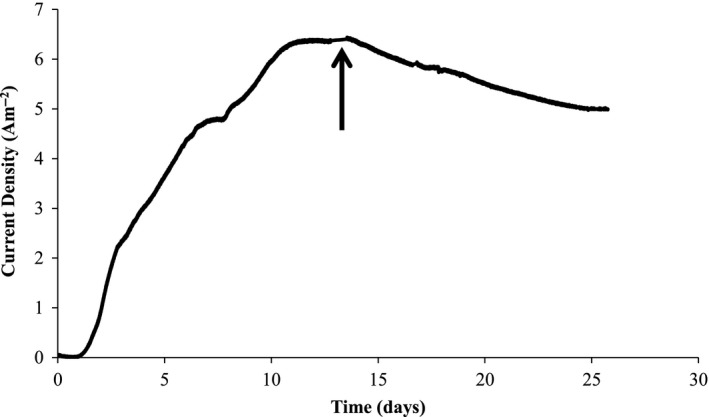
Chronoamperometry shows current generation (black line) from cellulose‐fed MEC 1 over 25 days. Black arrow indicates time (day 13) when LSCV was taken.

Chronoamperometry results in Fig. 1 indicate that a consortium of cellulose‐fermenting bacteria and ARB was capable of producing a sustained current density of 6.4 A m^−2^ in microbial electrolysis cell 1 (MEC 1) when using cellulose as the sole donor substrate. The corresponding coulombic efficiency (CE) and coulombic recovery (CR) were 84% and 46%, respectively, and were calculated on the final day (day 26). The chemical oxygen demand (COD) conversion rate was 0.05 gCOD l^−1^ day^−1^. The current density was much higher than previously reported for cellulose‐fed thermophilic microbial fuel cells (MFCs) at 0.4 A m^−2^ (Mathis *et al*., 2008), cellulose‐fed mesophilic MFCs at 0.05 A m^−2^ (Ren *et al*., 2008) and < 0.18 A m^−2^ (Rismani‐Yazdi *et al*., 2007) and cellulose‐fed mesophilic MECs at 1.8 A m^−2^ (Niessen *et al*., 2005). The higher current density in the MECs from this study in comparison to the MFCs from other studies was partly the result of the MEC mode of operation, which eliminated O_2_ intrusion to the anode and allowed the potential of the anode to be poised. At day 25, both anodes in MEC 1 were sacrificed for the purpose of imaging active biofilm anodes with scanning electron microscopy (SEM) and confocal laser scanning microscopy (CLSM).

**Figure 2:**
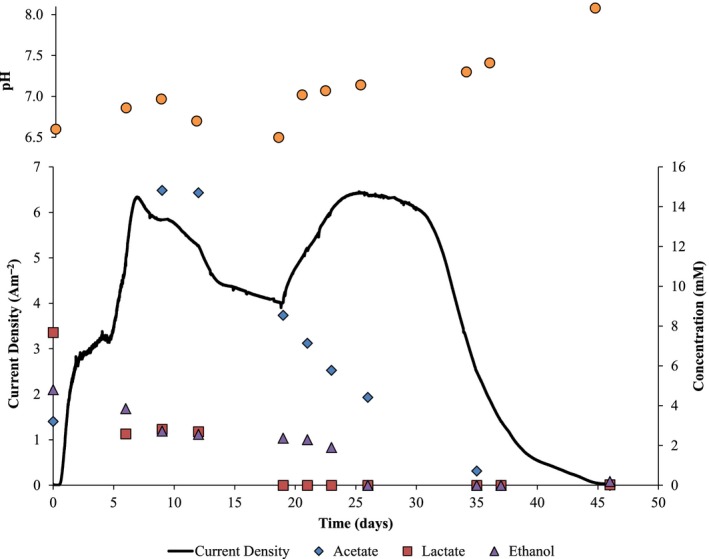
Current generation (black line) from cellulose‐fed MEC 2 along with concentrations of fermentation by‐products in mM is shown: acetate (blue diamonds), lactate (red squares) and ethanol (purple triangles). Corresponding pH is indicated by orange circles.

Batch bottle studies with cellulolytic cultures indicated that the four primary products formed from cellulose fermentation were acetate, lactate, ethanol and H_2_ (Fig. S1A–F). Previous studies have reported that cellulose fermentation products can be utilized in MxCs for the production of electricity or hydrogen using other mixed cultures (Niessen *et al*., 2005; Rismani‐Yazdi *et al*., 2007; Ren *et al*., 2008). To assess the products of cellulose fermentation and their role in current production in MECs, liquid samples were acquired during batch operation of MEC 2, and the results are in Fig. 2. The acetate concentration rose as cellulose fermentation occurred and then fell as it was consumed by ARB for current generation. The initial acetate concentration (2 mM) was from fermentation of cellulose in the serum bottles. However, as the filter paper was not completely degraded in the serum bottles before transferring to the MEC, an increasing acetate concentration (~15 mM) was the result of cellulose fermentation in the anode compartment.

In addition, pH measurements in MEC 2 indicated that acetate production led to a decrease in pH, while, following lactate depletion, diminishing acetate concentrations led to a rise in pH. The drop in pH between days 9 (pH = 7) and 20 (pH = 6.5) correlated with a decrease in current production, suggesting that the biofilm anode became pH‐inhibited (Torres *et al*., 2008; Marcus *et al*., 2011; Yuan *et al*., 2012; Lusk *et al*., 2015, 2016). (See Fig. S3 for confocal laser scanning microscopy confirming that the live portion of the biofilm was limited to 40–60 μm at the operating buffer concentration and anode configuration.) The small anode surface area in comparison to the bulk volume created a scenario in which fermentation products could accumulate faster than they were consumed, resulting in acidic conditions within the biofilm anode and bulk media.

Although H_2_ production was observed in all cellulolytic serum bottles (Fig. S1A–F), H_2_ was observed in neither MEC 1 nor MEC 2. Thermophilic ARB, including *T. ferriacetica* and *T. pseudethanolicus*, are capable of H_2_ consumption when performing dissimilatory metal reduction (Onyenwoke *et al*., 2007; Zavarzina *et al*., 2007); thus, it is likely that, if any H_2_ was produced during cellulose or ethanol fermentation in an MEC, it was quickly consumed by anode respiration. In addition, the ethanol concentration decreased gradually during MEC operation, whether acetate was increasing or decreasing; the decrease in ethanol was due to its fermentation to acetate or to its consumption by the biofilm anode. By day 37, lactate and ethanol concentrations became undetectable, and the acetate concentration was < 1 mM. However, biomass endogenous decay sustained current production at < 1 A m^−2^ for nine days. TCOD analysis indicated a CE of 84%, a CR of 62% and a COD conversion rate of 0.04 gCOD l^−1^ day^−1^. Previous reports attribute ~15% of non‐current electrons to net biomass synthesis (Rabaey *et al*., 2003; Lee *et al*., 2008; Li *et al*., 2015); therefore, most of the non‐recovered electrons possibly were contained in non‐decayed biomass in the biofilm anode.

CH_4_ gas was not detected in the anode of MEC 1 or MEC 2. As CH_4_ acts as an electron sink in MECs, inhibiting its production increases CE and CR, as more electrons can be recovered as current (Lee *et al*., 2008). The absence of CH_4_ despite the presence of 15 mM acetate means that either acetoclastic methanogenic archaea were absent or were inhibited in the thermophilic MECs for the duration of the experiments – an observation consistent with other studies (Nozhevnikova *et al*., 2007; Lü *et al*., 2014).

### Low‐scan rate cyclic voltammetry

**Figure 3:**
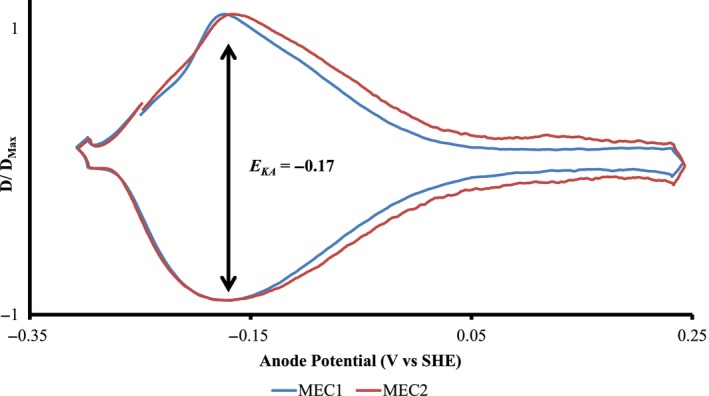
Derivative LSCV at 1 mV s^−1^ (normalized to D/D_M__ax_) from cellulose‐fed MECs 1 (day 13) (blue) and 2 (day 11) (red). Black arrows indicate the *E*_*ka*_.

The derivative LSCV plots (Fig. 3) reveal a mid‐point potential (*E*_*ka*_) of −0.169 ± 0.003 V versus SHE, and the same value occurred in a duplicate reactor. This *E*_*ka*_ value is similar to previously reported data for *T. pseudethanolicus* (Lusk *et al*., 2015), suggesting that the ARB from the enriched cellulolytic culture played a role in current production. Similar *E*_*ka*_ values from MEC 1 and MEC 2 indicate a consistent microbial consortium on the anode biofilm across duplicate reactors.

### Community analysis of thermophilic, cellulolytic microbial consortium

**Figure 4:**
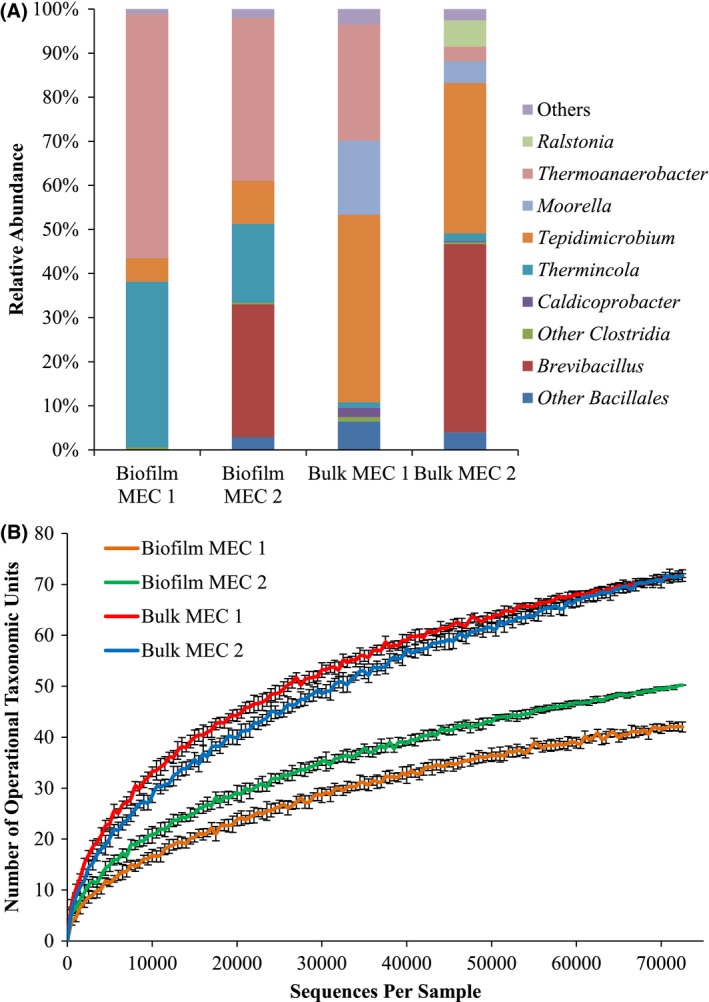
A. Relative abundance of bacterial genera for samples taken from either the bulk media (bulk) or the biofilm anode (biofilm) in MEC 1 and MEC 2. B. Rarefaction curve for ɑ‐diversity in the biofilm anode (biofilm MEC 1 is orange and biofilm MEC 2 is green) or bulk media (bulk MEC 1 is red and bulk MEC 2 is blue). Standard deviation is indicated by black bars.

The DNA‐sequence analysis in Fig. 4A shows that biofilm anodes contained a greater abundance of bacterial genera associated with ARB and dissimilatory metal reduction than the bulk medium, because these bacteria may require the anode for respiration. Results indicate that the biofilm anode was enriched with bacteria from the *Thermincola* genus (28 ± 14%, turquoise in Fig. 4A), representing the *Thermincola ferriacetica* ARB that was used to inoculate the MECs. The presence of the *Thermoanaerobacter* genus (pink in Fig. 4A) is important in the biofilm anode (46 ± 13%) and the anode bulk medium (26% in Bulk MEC 1 and 3% in Bulk MEC 2), as *T. pseudethanolicus* has been implicated in previous studies for its ability to ferment cellulose fermentation products including glucose and cellobiose while simultaneously performing current production in an MEC (Lusk *et al*., 2015).

Another major inhabitant of the biofilm anode was the *Tepidmicrobium* (7.6 ± 3.1%) genus from the Tissierellaceae family (orange in Fig. 4A), which also made up a large portion of the anode bulk medium (38 ± 5.9%). Members of the *Tepidmicrobium* genus, including *Tepidmicrobium ferriphilum* and *Tepidmicrobium xylanilyticum*, have been reported as capable of oxidizing proteinaceous substrates or carbohydrates while simultaneously reducing either 9,10‐anthraquinone 2,6‐disulfonate (AQDS) or Fe(III) oxides (Slobodkin *et al*., 2006; Niu *et al*., 2009). It is likely that this genus was selected for in the cellulolytic fermentation bottles and then functioned as a fermenter and an ARB once it was inoculated into the MECs.

In contrast to ARB, the cellulolytic microbial consortium responsible for fermentation occupied a higher percentage of the bulk medium community and was enriched. *Moorella* was most abundant in the bulk medium of MEC 1 (17%) and made up 5% of the bulk medium in MEC 2. *Moorella* species, including *Moorella thermoacetica*, have been reported to convert the products of cellulose degradation into acetate in batch reactors (Savage and Drake, 1986; Hu *et al*., 2013). *Caldicoprobacter* made up ~2.4% of the bulk media in MEC 1 (dark purple in Fig. 4A). Members of this genus, including *Caldicoprobacter oshimai* and *Caldicoprobacter algeriensis*, are thermophilic, cellulolytic, xylanolytic and fermentative (Yokoyama *et al*., 2010; Bouanane‐Darenfed *et al*., 2011). In addition, members of the *Clostridium* genus (dark green in Fig. 4A) were present in the bulk medium (0.7 ± 0.5%) and biofilm anode (0.5 ± 0.1%). Members of the *Clostridium* genus, including *Clostridium thermocellum*, produce cellulosomes and are well documented as cellulolytic thermophiles (Viljoen *et al*., 1926; Akinosho *et al*., 2014).

The *Brevibacillus* genus (red in Fig. 4A) was the most variable. In MEC 1, *Brevibacillus* was not present in the biofilm anode and made up only ~0.02% of the bulk medium; however, it was highly abundant in the biofilm anode (30%) and bulk media (43%) of MEC 2. *Brevibacillus*, including *Brevibacillus* sp. strain JXL and *Brevibacillus laterosporus*, has been reported to produce cellulosomes (Kato *et al*., 2005; Liang *et al*., 2009). In addition, *Ralstonia* (light green in Fig. 4A), a gram‐negative bacterial genus, was present in the bulk media of MEC 2 (6%). Members of the *Ralstonia* genus, including *Ralstonia paucula*, have been identified in mixed thermophilic lipolytic cultures (Sheikh Abdul Hamid *et al*., 2003). It is likely that *Brevibacillus* and *Ralstonia* were key microbial players for cellulolytic activity and microbial decay in MEC 2 as samples were taken after the current density had reached ~0.0 A m^−2^.

The rarefaction plot in Fig. 4B shows that within‐reactor microbial communities present in the bulk media (Bulk MEC 1 and Bulk MEC 2) were much more diverse than the microbial communities composing the biofilm anode (biofilm MEC 1 and biofilm MEC 2). However, between reactors, the biofilm anodes and microbial communities present in the bulk media of MECs 1 and 2 had similar sequence diversity, supporting that the composition of the enriched cellulolytic community is repeatable and robust.

### Scanning electron microscopy

**Figure 5:**
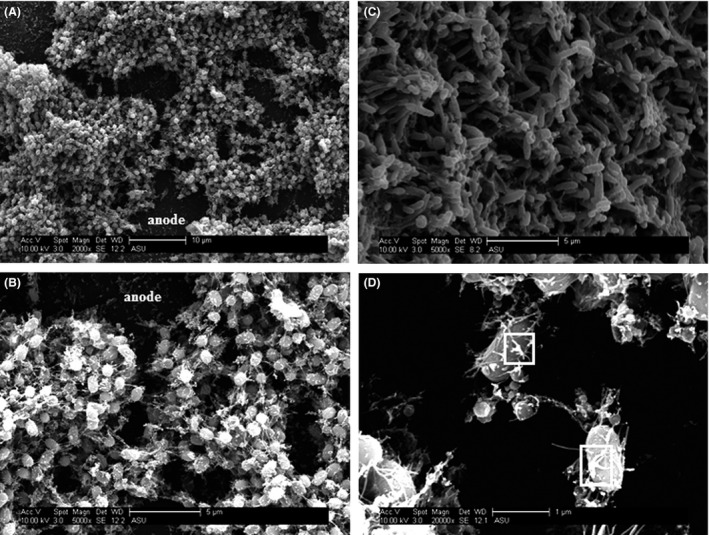
Scanning electron microscopy images reveal a biofilm with diverse bacterial morphologies. A. Shows an active biofilm anode attached to an electrode at 2000× magnification. The surface of the graphite anode is indicated by the word ‘anode’. B. An active biofilm anode attached to an electrode at 5000× magnification. The surface of the graphite anode is indicated by the word ‘anode’. C. An active biofilm anode attached to an electrode at 5000× magnification. D. An active biofilm anode attached to an electrode at 20 000× magnification. The white squares are used to highlight features present on the cell surface.

Scanning electron microscopy analysis revealed biofilm anodes composed of a diverse set of cell morphologies. The medium rod‐shaped cells (Fig. 5A–C) are indicative of clostridial bacteria (Zavarzina *et al*., 2007; Parameswaran *et al*., 2013). Also present are bacteria containing long, rod‐shaped structures with spore‐like appendages (white boxes in Fig. 5D), which may indicate the presence of cellulosomes (Freier *et al*., 1988).

## Experimental procedures

### Growth and media conditions for thermophilic anode‐respiring bacteria

*Thermincola ferriacetica* strain 14005 was obtained from DSMZ, Braunshweig, Germany. The strain was cultivated in serum bottles containing a modified DSMZ Medium 962: *Thermovenabulum* medium. The contents of the medium can be found in the Supporting Information. The cultures were grown in 160‐ml batch serum bottles containing 100 ml medium and were incubated in an Excella E24 Incubator Shaker (New Brunswick Scientific) at 60°C and 150 RPM.

### Growth conditions and medium for enrichment of cellulolytic bacterial consortium

An enriched cellulolytic consortium including *Thermoanaerobacter pseudethanolicus* was cultivated in serum bottles using ATCC Medium 1190. The contents of the medium can be found in the Supporting Information. The cultures were grown in 160‐ml batch serum bottles containing 100‐ml medium and were incubated in an Excella E24 Incubator Shaker (New Brunswick Scientific) at 60°C and 150 RPM. Degradation of cellulose was monitored visually, and the products of cellulose fermentation were monitored via high pressure liquid chromatography (HPLC). Cultures from serum bottles showing the highest affinity for cellulose degradation were transferred (3 ml) to new serum bottles containing fresh media and cellulose. Due to increased rates of cellulose fermentation (Fig. S1A–F), the contents of bottles containing filter paper (Fig. S1D and E) were added to the microbial electrochemical cells after glucose concentrations had diminished.

### Construction, operation and monitoring of dual‐chamber H‐type microbial electrolysis cells

Duplicate H‐type microbial electrochemical cells (MECs) (Fig. S2) were constructed containing a 350‐ml anode chamber and a 350‐ml cathode chamber for a total reactor volume of 700 ml (Parameswaran *et al*., 2013; Lusk *et al*., 2016). An anion exchange membrane (AMI 7001; Membranes International, Glen Rock, NJ, USA) was used to allow ion transfer between the anode and cathode. The anode electrodes were comprised of two graphite rods (0.3 cm diameter, graphitestore.com) with a total anode surface area of 2.40 and 2.87 cm^2^. An Ag/AgCl reference electrode (BASi MF‐2052) was placed in the anode chamber. The anode was poised at −0.06 V versus the standard hydrogen electrode (SHE) using a potentiostat (Princeton Applied Research, Model VMP3, Oak Ridge, TN, USA). The anode chambers were kept completely mixed via agitation from a magnetic stir bar operated at 200 rpm. The cathode consisted of a single cylindrical graphite rod (0.3 cm diameter and a total area of 6.67 cm^2^). Cathode pH was adjusted to 12 via addition of NaOH. Gas collection bags were placed on the anode compartments to collect volatile products (CO_2_) and on the cathode to collect hydrogen.

Anode chambers were inoculated from serum bottles containing an enriched cellulolytic culture containing *T. pseudethanolicus* and with a separate culture of *T. ferriacetica*. For the enriched cellulolytic culture, after 10 days of growth (glucose concentration = 0 mM), 200 ml of spent ATCC Medium 1190 containing the cellulolytic culture with visibly unfermented filter paper was transferred in a glove box (Coy) under anaerobic conditions (mix of 97.5% UHP N_2_ and 2.5% UHP H_2_) to the anode chamber of an H‐type MEC. In addition, 150 ml of modified DSMZ Medium 962 media without acetate and 3 ml of *T. ferriacetica* from stock serum bottles (~10 days of growth) were added to the anode of the MEC under anaerobic conditions. The MECs were operated in batch mode in a 60°C incubator.

ec‐lab software (version 10.31) was used to constantly monitor current in two minute intervals during chronoamperometry (CA) and to observe the *j‐V* response of the biofilm anode during low‐scan cyclic voltammetry (LSCV). LSCV scans were performed 1 and 10 mV s^−1^. Derivative values were obtained using the Derivative Process with the ec‐lab software.

### Monitoring cell performance via analysis of fermentation products with high pressure liquid chromatography, total chemical oxygen demand and gas chromatography

To monitor the fermentation of cellulose, the consumption of fermentation products and pH, 1 ml of samples was taken from the serum bottles daily and from the MEC reactors every ~4 days. The pH was monitored using an Orion 2 Star pH Benchtop apparatus (Thermoscientific, Waltham, MA, USA). Liquid samples were filtered through a 0.2 μm filter and stored at −20°C until they were analysed using HPLC (Shimadzu, Kyoto, Japan) via the method from (Lusk *et al*., 2015). Fermentation products monitored included acetate, lactate, butyrate, ethanol, glucose and cellobiose.

Initial and final concentrations of total chemical oxygen demand (TCOD) were measured using a Hach 20–1500 mg l^−1^ range TCOD kit (this measurement accounted for initial and final suspended biomass). A biochemical methane potential (BMP) test was used on the yeast extract to determine its potential as an electron source, as *T. ferriacetica* growth on yeast extract has been reported previously (Zavarzina *et al*., 2007). The BMP test indicated that yeast extract had minimal influence on current production with an equivalent of 19 mA*h per reactor. TCOD measurements were used to calculate CE (Parameswaran *et al*., 2013) CR (Ge *et al*., 2013). CE measures the conversion efficiency of the electrons removed from the donor substrate and utilized for current production, while CR compared the current production to the total electrons entering the MEC (Lee *et al*., 2008).

Coulombic efficiency can be calculated as:

(1) $$\text{CE}=\frac{{\text{TCOD}}_{\mathrm{current}}}{{\text{TCOD}}_{\mathrm{initial}}-{\text{TCOD}}_{\mathrm{final}}},$$

where TCOD_current_ is a measure of the number of coulombs captured as current, TCOD_final_ is the total number of coulombs measured as TCOD contained in the bulk liquid after the batch run, and TCOD_initial_ is the total number of coulombs measured as TCOD contained in the cellulose and bulk media prior to the batch run.

Coulombic recovery can be calculated as:

(2) $$\text{CR}=\frac{{\text{TCOD}}_{\mathrm{current}}}{{\text{TCOD}}_{\mathrm{initial}}},$$

where TCOD_current_ is a measure of the number of coulombs captured as current, and TCOD_initial_ is the total number of coulombs measured as TCOD contained in the cellulose and bulk media prior to the batch run.

Gas production in the headspace of serum bottles and MECs were periodically measured using a frictionless glass syringe (Perfektum, NY, USA). For serum bottles, gas for measurements was collected directly from the headspace of the bottles, and for the MECs, gas for measurements was collected from 0.5 l gas collection bags (Fisher Scientific, Waltham, MA, USA). Gas samples were collected using a gas‐tight syringe (500 μl, Trajan Scientific, Melbourne, Australia). H_2_ and CO_2_ production were quantified using a gas chromatograph (GC 2010; Shimadzu) equipped with a thermal conductivity detector and a packed column (ShinCarbon ST 100/120 mesh, Restek Corporation, Bellefonte, PA, USA) for separating sample gases. N_2_ was the carrier gas fed at a constant flow rate of 10 ml min^−1^, and the temperature conditions for injection, column and detector were 110, 140 and 160°C respectively. Analytical grade H_2_, CH_4_ and CO_2_ were used for standard calibration curves. Detection limits of H_2_ and CH_4_ were 0.5% (or 2.5 ml in a 500 ml bag; the theoretical maximum quantity of undetected H_2_ and CH_4_ about 0.21 mg and 1.6 mg respectively). CH_4_ was monitored, but it was not observed in any MECs or serum bottles. H_2_ was observed in all serum bottles, but was not observed in the gas phase of the anode in any MECs.

### Confocal laser scanning microscopy (CLSM) and SEM

Microscopy measurements were completed by sacrificing live biofilms from an MEC after its current had reached a steady state. To ascertain the thickness of the live and dead biomass on the anode, we employed confocal laser scanning microscopy (CSLM) using the LIVE/DEAD stain (BacLight Cell vitality kit; Invitrogen, Carlsbad, CA, USA) applied to intact biofilms connected to anodes (Parameswaran *et al*., 2013; Lusk *et al*., 2015, 2016). Measurements were acquired using an upright Leica SP5 microscope. Images were taken every 5 μm with a 40X immersion objective (Images shown in Fig. S3).

Scanning electron microscopy also was performed on an intact biofilms connected to anodes. After its removal from the reactor, the biofilm anode was fixed with 4% glutaraldehyde for 12 h at 4°C and then washed and stored in 10‐mM PBS (pH 7) solution for ~1 h. Next, the sample was then treated with 1% osmium tetroxide for 15 min, followed by graded ethanol series dehydration (50%, 70%, 95%, and 100% for 5 min each), then dried by critical point drying and, finally, mounted on an aluminium stub before being sputter‐coated with a Au/Pd alloy with a Technics Hummer II sputter coater. Imaging was conducted using an FEI XL‐30 environmental SEM (Philips) with an accelerating voltage of 10–20 kV and a working distance of 8–10 mm.

### DNA extraction and pyrosequencing community analysis

For bacterial community analysis of the biofilm anodes, biofilms were collected in sterilized 1.5‐ml centrifuge vials at the end of each run (day 26 for MEC 1 and day 46 for MEC 2). For bacterial community analysis of the bulk media, 150 ml of liquid was removed and placed in three sterilized 50 ml Falcon tubes per reactor. The tubes were then centrifuged (5810 R, Eppendorf, Hamburg, Germany) at 4000 RPM for 15 min, and the pellets were preserved. All DNA collection was conducted at the end of the MEC batch runs. DNA extraction was performed using the gram‐positive bacteria method from the Qiagen DNEasy Blood and Tissue Extraction Kit (Qiagen, Mississauga, ON, USA) following the manufacturer's recommendations. DNA extraction was confirmed and quantified using a Nanodrop ND‐1000 spectrophotometer. Extracted DNA was stored at −20°C.

Amplicon pyrosequencing was conducted at the Microbiome Analysis Lab (Arizona, USA) using a MiSeq Ilumina sequencer. The V4 region of the 16S rRNA gene was targeted with barcode primers 515f and 806r to analyse the bacterial domain. Raw data were scrutinized using qiime 1.4.0 suite (Walters *et al*., 2010): Sequences having < 200 bps, homopolymers > 6 bps, primer mismatches or an average quality score < 25 were removed. The Greengenes 16S rRNA gene database with *uclust* (Edgar, 2010) was used to pick the operational taxonomic unit (OTU) based on ≥ 97% identity. OTUs that contained < two sequences (singletons) were removed. Remaining OTUs were aligned with the representative sequence in the Greengenes database using *PyNast* (DeSantis *et al*., 2006; Caporaso *et al*., 2010). chimeraslayer was used to identify chimeric sequences (Haas *et al*., 2011), which were removed using a python script in qiime. OTUs were assigned a taxonomy using a 50% confidence threshold with the ribosomal database project (RDP) (Wang *et al*., 2007). Whole‐tree phylogenetic diversity was analysed with qiime 1.4.0 (Walters *et al*., 2010).

## Outlook

Operating thermophilic MxCs for current production from cellulose degradation shows consistently high current density, CE and CR in the absence of CH_4_ production. However, the operating temperature, bacterial consortia and dimensional properties of thermophilic MxCs will play a crucial role in further development of the technology for consolidated bioprocessing. For example, previous reports indicate that optimal activity for *T. ferriacetica*, one of the primary ARB in this study, is 60°C (Zavarzina *et al*., 2007; Mathis *et al*., 2008; Marshall and May, 2009; Parameswaran *et al*., 2013); however, *T. pseudethanolicus* has optimal activity at a higher temperature (65–70°C) (Onyenwoke *et al*., 2007). In addition, reports indicate that cellulase activity may be optimal in other thermophilic bacteria at temperatures higher than 60°C (Johnson *et al*., 1982; Curatolo *et al*., 1983; Blumer‐Schuette *et al*., 2012; Basen *et al*., 2014). Given that many dissimilatory metal‐reducing thermophiles could be ARB, it may be possible to increase current production via fermentation product utilization by employing additional microorganisms not observed in this study (Roh *et al*., 2002; Slobodkin *et al*., 2006; Niu *et al*., 2009; Slepova *et al.,* 2009). Previous reports also indicated that anode surface area should be optimized to account for the rate of production of acids and alcohols from cellulose fermentation which may increase the COD conversion rate (Logan *et al*., 2007; Mathis *et al*., 2008). Future research directions should focus on a holistic view of cellulolytic MxCs by analysing the kinetics of cellulase activity, fermentation product production and consumption, anode respiration, bacterial growth and nutrient balancing.

## Conflict of interest

The authors declare no competing financial interest.

## Supporting information


**Fig. S1**. (a–f) Representative fermentation profiles tracked over 11 days from six serum bottles.
**Fig. S2**. Picture of a thermophilic H‐type microbial electrochemical cell in an incubator at 60 °C.
**Fig. S3**. CLSM LIVE/DEAD analysis revealed a live biofilm layer (*Lf* shown in yellow) approximately 40‐60 μm thick.

## References

[mbt212733-bib-0001] Akinosho, H. , Yee, K. , Close, D. , and Ragauskas, A. (2014) The emergence of *Clostridium thermocellum* as a high utility candidate for consolidated bioprocessing applications. Front Chem 2: 66. 25207268 10.3389/fchem.2014.00066 PMC4143619

[mbt212733-bib-0002] Badger, P.C. (2002) Processing Cost Analysis for Biomass Feedstock. Prepared for the US Department of Energy. Under Contract DE‐AC05‐00OR22725. *ORNL. U. S. Atomic Energy Commission, TM‐2002(199)*.

[mbt212733-bib-0003] Basen, M. , Rhaesa, A. , Kataeva, I. , Prybol, C. , Scott, I. , Poole, F. , and Adams, M. (2014) Degradation of high loads of crystalline cellulose and of unpretreated plant biomass by the thermophilic bacterium *Caldicellulosiruptor bescii* . Bioresour Technol 152: 384–392. 24316482 10.1016/j.biortech.2013.11.024

[mbt212733-bib-0004] Blumer‐Schuette, S.E. , Giannone, R.J. , Zurawski, J.V. , Ozdemir, I. , Ma, Q. , Yin, Y. , *et al* (2012) *Caldicellulosiruptor* core and pangenomes reveal determinants for noncellulosomal thermophilic deconstruction of plant biomass. J Bacteriol 194: 4015–4028. 22636774 10.1128/JB.00266-12 PMC3416521

[mbt212733-bib-0005] Bouanane‐Darenfed, A. , Fardeau, M.L. , Grégoire, P. , Joseph, M. , Kebbouche‐Gana, S. , Benayad, T. , *et al* (2011) *Caldicoprobacter algeriensis* sp. nov. a new thermophilic anaerobic, xylanolytic bacterium isolated from an Algerian hot spring. Curr Microbiol 62: 826–832. 20981546 10.1007/s00284-010-9789-9

[mbt212733-bib-0006] Bryant, C. (2011) Putting the pieces together, cellulosic commercialization. National Ethanol Conference.

[mbt212733-bib-0007] Caporaso, J.G. , Bittinger, K. , Bushman, F.D. , DeSantis, T.Z. , Andersen, G.L. , and Knight, R. (2010) PyNAST: a flexible tool for aligning sequences to a template alignment. Bioinformatics 26: 266–267. 19914921 10.1093/bioinformatics/btp636 PMC2804299

[mbt212733-bib-0008] Carere, C.R. , Sparling, R. , Cicek, N. , and Levin, D.B. (2008) Third generation biofuels via direct cellulose fermentation. Int J Mol Sci 9: 1342–1360. 19325807 10.3390/ijms9071342 PMC2635718

[mbt212733-bib-0009] Curatolo, W. , Kanodia, S. , and Roberts, M.F. (1983) The effect of ethanol on the phase behavior of membrane lipids extracted from *Clostridium thermocellum* strains. BBA –. Biomembranes 734: 336–341.

[mbt212733-bib-0010] Demain, A.L. , Newcomb, M. , and David Wu, J.H. (2005) Cellulase, clostridia, and ethanol. Microbiol Mol Biol Rev 69: 124–154. 15755956 10.1128/MMBR.69.1.124-154.2005 PMC1082790

[mbt212733-bib-0011] DeSantis, T.Z. , Hugenholtz, P. , Larsen, N. , Rojas, M. , Brodie, E.L. , Keller, K. , *et al* (2006) Greengenes, a chimera‐checked 16S rRNA gene database and workbench compatible with ARB. Appl Environ Microbiol 72: 5069–5072. 16820507 10.1128/AEM.03006-05 PMC1489311

[mbt212733-bib-0012] Edgar, R.C. (2010) Search and clustering orders of magnitude faster than BLAST. Bioinformatics 26: 2460–2461. 20709691 10.1093/bioinformatics/btq461

[mbt212733-bib-0013] Emtiazi, G. , and Nahvi, I. (2000) Multi‐enzyme production by *Cellulomonas* sp. grown on wheat straw. Biomass Bioenerg 19: 31–37.

[mbt212733-bib-0014] Freier, D. , Mothershed, C.P. , and Wiegel, J. (1988) Characterization of *Clostridium thermocellum* JW20. Appl Environ Microbiol 54: 204–211. 16347527 10.1128/aem.54.1.204-211.1988 PMC202422

[mbt212733-bib-0015] Ge, Z. , Ping, Q. , Xiao, L. , and He, Z. (2013) Reducing effluent discharge and recovering bioenergy in an osmotic microbial fuel cell treating domestic wastewater. Desalination 312: 52–59.

[mbt212733-bib-0016] Haas, B.J. , Gevers, D. , Earl, A.M. , Feldgarden, M. , Ward, D.V. , Giannoukos, G. , *et al* , and Human Microbiome Consortium, The Human Microbiome Consortium (2011) Chimeric 16S rRNA sequence formation and detection in Sanger and 454‐pyrosequenced PCR amplicons. Genome Res 21: 494–504. 21212162 10.1101/gr.112730.110 PMC3044863

[mbt212733-bib-0017] Hama, S. , Nakano, K. , Onodera, K. , Nakamura, M. , Noda, H. , and Kondo, A. (2014) Saccharification behavior of cellulose acetate during enzymatic processing for microbial ethanol production. Bioresour Technol 157: 1–5. 24514162 10.1016/j.biortech.2014.01.002

[mbt212733-bib-0018] Hu, P. , Rismani‐Yazdi, H. , and Stephanopoulos, G. (2013) Anaerobic CO_2_ fixation by the acetogenic bacterium *Moorella thermoacetica* . AIChE J 59: 3176–3183.

[mbt212733-bib-0019] Johnson, E.A. , Sakajoh, M. , Halliwell, G. , Madia, A. , and Demain, A.L. (1982) Saccharification of complex cellulosic substrates by the cellulase system from *Clostridium thermocellum* . Appl Environ Microbiol 43: 1125–1132. 16346009 10.1128/aem.43.5.1125-1132.1982 PMC244196

[mbt212733-bib-0020] Kato, S. , Haruta, S. , Cui, Z.J. , Ishii, M. , and Igarashi, Y. (2005) Stable coexistence of five bacterial strains as a cellulose‐degrading community. Appl Environ Microbiol 71: 7099–7106. 16269746 10.1128/AEM.71.11.7099-7106.2005 PMC1287685

[mbt212733-bib-0021] Kim, J.R. , Jung, S.H. , Regan, J.M. , and Logan, B.E. (2007) Electricity generation and microbial community analysis of alcohol powered microbial fuel cells. Bioresour Technol 98: 2568–2577. 17097875 10.1016/j.biortech.2006.09.036

[mbt212733-bib-0022] Lee, H. , Parameswaran, P. , Kato‐Marcus, A. , Torres, C.I. , and Rittmann, B.E. (2008) Evaluation of energy‐conversion efficiencies in microbial fuel cells (MFCs) utilizing fermentable and non‐fermentable substrates. Water Res 42: 1501–1510. 18035391 10.1016/j.watres.2007.10.036

[mbt212733-bib-0023] Li, H. , Knutson, B.L. , Nokes, S.E. , Lynn, B.C. , and Flythe, M.D. (2012) Metabolic control of *Clostridium thermocellum* via inhibition of hydrogenase activity and the glucose transport rate. Appl Microbiol Biotechnol 93: 1777–1784. 22218768 10.1007/s00253-011-3812-3

[mbt212733-bib-0024] Li, N. , Kakarla, R. , Moon, J.M. , and Min, B. (2015) Determination of microbial growth by protein assay in an air‐cathode single chamber microbial fuel cell. J Microbiol Biotechnol 25: 1114. 25674807 10.4014/jmb.1412.12067

[mbt212733-bib-0025] Liang, Y. , Yesuf, J. , Schmitt, S. , Bender, K. , and Bozzola, J. (2009) Study of cellulases from a newly isolated thermophilic and cellulolytic *Brevibacillus sp*. strain JXL. J Ind Microbiol Biotechnol 36: 961–970. 19390881 10.1007/s10295-009-0575-2

[mbt212733-bib-0026] Logan, B. , Cheng, S. , Watson, V. , and Estadt, G. (2007) Graphite fiber brush anodes for increased power production in air‐cathode microbial fuel cells. Environ Sci Technol 41: 3341–3346. 17539547 10.1021/es062644y

[mbt212733-bib-0027] Lü, F. , Bize, A. , Guillot, A. , Monnet, V. , Madigou, C. , Chapleur, O. , *et al* (2014; 2013) Metaproteomics of cellulose methanisation under thermophilic conditions reveals a surprisingly high proteolytic activity. ISME J 8: 88–102. 23949661 10.1038/ismej.2013.120 PMC3869005

[mbt212733-bib-0028] Lusk, B.G. , Khan, Q.F. , Parameswaran, P. , Hameed, A. , Ali, N. , Rittmann, B.E. , and Torres, C.I. (2015) Characterization of electrical current‐generation capabilities from thermophilic bacterium *Thermoanaerobacter pseudethanolicus* using xylose, glucose, cellobiose, or acetate with fixed anode potentials. Environ Sci Technol 49: 14725. 26569143 10.1021/acs.est.5b04036

[mbt212733-bib-0029] Lusk, B.G. , Parameswaran, P. , Popat, S.C. , Rittmann, B.E. , and Torres, C.I. (2016) The effect of pH and buffer concentration on anode biofilms of *Thermincola ferriacetica* . Bioelectrochemistry 112: 47–52. 27450427 10.1016/j.bioelechem.2016.07.007

[mbt212733-bib-0030] Lynd, L.R. , Weimer, P.J. , van Zyl, W.H. , and Pretorius, I.S. (2002) Microbial cellulose utilization: fundamentals and biotechnology. Microbiol Mol Biol Rev 66: 506–577. 12209002 10.1128/MMBR.66.3.506-577.2002 PMC120791

[mbt212733-bib-0031] Marcus, A.K. , Torres, C.I. , and Rittmann, B.E. (2011) Analysis of a microbial electrochemical cell using the proton condition in biofilm (PCBIOFILM) model. Bioresour Technol 102: 253–262. 20395137 10.1016/j.biortech.2010.03.100

[mbt212733-bib-0032] Marshall, C.W. , and May, H.D. (2009) Electrochemical evidence of direct electrode reduction by a thermophilic Gram‐positive bacterium, Thermincola ferriacetica. Energy Environ Sci 2: 699–705.

[mbt212733-bib-0033] Mathis, B.J. , Marshall, C.W. , Milliken, C.E. , Makkar, R.S. , Creager, S.E. , and May, H.D. (2008) Electricity generation by thermophilic microorganisms from marine sediment. Appl Microbiol Biotechnol 78: 147–155. 18080121 10.1007/s00253-007-1266-4

[mbt212733-bib-0034] McBee, R.H. (1950) The anaerobic thermophilic cellulolytic bacteria. Bacteriol Rev 14: 51–63. 15420123 10.1128/br.14.1.51-63.1950 PMC440954

[mbt212733-bib-0035] Niessen, J. , Schröder, U. , Harnisch, F. , and Scholz, F. (2005) Gaining electricity from in situ oxidation of hydrogen produced by fermentative cellulose degradation. Lett Appl Microbiol 41: 286–290. 16108922 10.1111/j.1472-765X.2005.01742.x

[mbt212733-bib-0036] Niu, L. , Song, L. , Liu, X. , and Dong, X. (2009) *Tepidimicrobium xylanilyticum* sp. nov., an anaerobic xylanolytic bacterium, and emended description of the genus *Tepidimicrobium* . Int J Syst Evol Microbiol 59: 2698–2701. 19625437 10.1099/ijs.0.005124-0

[mbt212733-bib-0037] Nozhevnikova, A.N. , Nekrasova, V. , Ammann, A. , Zehnder, A.J.B. , Wehrli, B. , and Holliger, C. (2007) Influence of temperature and high acetate concentrations on methanogenensis in lake sediment slurries. FEMS Microbiol Ecol 62: 336–344. 17949433 10.1111/j.1574-6941.2007.00389.x

[mbt212733-bib-0038] Oh, S. , and Logan, B.E. (2005) Hydrogen and electricity production from a food processing wastewater using fermentation and microbial fuel cell technologies. Water Res 39: 4673–4682. 16289673 10.1016/j.watres.2005.09.019

[mbt212733-bib-0501] Olson, D.G. , McBride, J.E. , Shaw, A.J. , and Lynd, L.R. (2012) Recent progress in consolidated bioprocessing. Curr Opin Biotechnol 23: 396–405. 22176748 10.1016/j.copbio.2011.11.026

[mbt212733-bib-0039] Onyenwoke, R.U. , Kevbrin, V.V. , Lysenko, A.M. , and Wiegel, J. (2007) *Thermoanaerobacter pseudethanolicus* sp. nov., a thermophilic heterotrophic anaerobe from Yellowstone National Park. Int J Syst Evol Microbiol 57: 2191–2193. 17911280 10.1099/ijs.0.65051-0

[mbt212733-bib-0040] Parameswaran, P. , Bry, T. , Popat, S.C. , Lusk, B.G. , Rittmann, B.E. , and Torres, C.I. (2013) Kinetic, electrochemical, and microscopic characterization of the thermophilic, anode‐respiring bacterium *Thermincola ferriacetica* . Environ Sci Technol 47: 4934–4940. 23544360 10.1021/es400321c

[mbt212733-bib-0041] Rabaey, K. , Lissens, G. , Siciliano, S.D. , and Verstraete, W. (2003) A microbial fuel cell capable of converting glucose to electricity at high rate and efficiency. Biotechnol Lett 25: 1531. 14571978 10.1023/a:1025484009367

[mbt212733-bib-0042] Ren, Z. , Steinberg, L.M. , and Regan, J.M. (2008) Electricity production and microbial biofilm characterization in cellulose‐fed microbial fuel cells. Water Sci Technol 58: 617–622. 18725730 10.2166/wst.2008.431

[mbt212733-bib-0043] Rismani‐Yazdi, H. , Christy, A.D. , Dehority, B.A. , Morrison, M. , Yu, Z. , and Tuovinen, O.H. (2007) Electricity generation from cellulose by rumen microorganisms in microbial fuel cells. Biotechnol Bioeng 97: 1398–1407. 17274068 10.1002/bit.21366

[mbt212733-bib-0044] Roh, Y. , Liu, S.V. , Li, G. , Huang, H. , Phelps, T.J. , and Zhou, J. (2002) Isolation and characterization of metal‐reducing *Thermoanaerobacter* strains from deep subsurface environments of the Piceance Basin, Colorado. Appl Environ Microbiol 68: 6013–6020. 12450823 10.1128/AEM.68.12.6013-6020.2002 PMC134454

[mbt212733-bib-0045] Saripan, A. , and Reungsang, A. (2014) Simultaneous saccharification and fermentation of cellulose for bio‐hydrogen production by anaerobic mixed cultures in elephant dung. Int J Hydrogen Energy 39: 9028–9035.

[mbt212733-bib-0046] Savage, M.D. , and Drake, H.L. (1986) Adaptation of the acetogen *Clostridium thermoautotrophicum* to minimal medium. J Bacteriol 165: 315–318. 3941046 10.1128/jb.165.1.315-318.1986 PMC214409

[mbt212733-bib-0047] Sheikh Abdul Hamid, N. , Zen, H.B. , Tein, O.B. , Halifah, Y.M. , Saari, N. , and Bakar, F.A. (2003) Screening and identification of extracellular lipase‐producing thermophilic bacteria from a Malaysian hot spring. World J Microbiol Biotechnol 19: 961–968.

[mbt212733-bib-0048] Sizova, M.V. , Izquierdo, J.A. , Panikov, N.S. , and Lynd, L.R. (2011) Cellulose‐ and xylan‐degrading thermophilic anaerobic bacteria from biocompost. Appl Environ Microbiol 77: 2282–2291. 21317267 10.1128/AEM.01219-10 PMC3067422

[mbt212733-bib-0500] Slepova, T.V. , Sokolova, T.G. , Kolganova, T.V. , Tourova, T.P. , and Bonch‐Osmolovskaya, E.A. (2009) Carboxydothermus siderophilus sp. nov., a thermophilic, hydrogenogenic, carboxydotrophic, dissimilatory Fe(III)‐reducing bacterium from a Kamchatka hot spring. Int J Syst Evol Microbiol 59: 213–217. 19196756 10.1099/ijs.0.000620-0

[mbt212733-bib-0049] Slobodkin, A.I. , Tourova, T.P. , Kostrikina, N.A. , Lysenko, A.M. , German, K.E. , Bonch‐Osmolovskaya, E.A. , and Birkeland, N. (2006) *Tepidimicrobium ferriphilum gen. nov., sp*. nov., a novel moderately thermophilic, Fe(III)‐reducing bacterium of the order Clostridiales. Int J Syst Evol Microbiol 56: 369–372. 16449442 10.1099/ijs.0.63694-0

[mbt212733-bib-0050] Taylor, M.P. , Eley, K.L. , Martin, S. , Tuffin, M.I. , Burton, S.G. , and Cowan, D.A. (2009) Thermophilic ethanologenesis: future prospects for second‐generation bioethanol production. Trends Biotechnol 27: 398–405. 19481826 10.1016/j.tibtech.2009.03.006

[mbt212733-bib-0051] Torres, C.I. , Kato Marcus, A. , and Rittmann, B.E. (2008) Proton transport inside the biofilm limits electrical current generation by anode‐respiring bacteria. Biotechnol Bioeng 100: 872–881. 18551519 10.1002/bit.21821

[mbt212733-bib-0052] Viljoen, J.A. , Fred, E.B. , and Peterson, W.H. (1926) The fermentation of cellulose by thermophilic bacteria. J Agri Sci 16: 1–17.

[mbt212733-bib-0053] Walters, W.A. , Pirrung, M. , Peña, A.G. , Huttley, G.A. , Zaneveld, J. , Kuczynski, J. , *et al* (2010) QIIME allows analysis of high‐throughput community sequencing data. Nat Methods 7: 335–336. 20383131 10.1038/nmeth.f.303 PMC3156573

[mbt212733-bib-0054] Wang, Q. , Garrity, G.M. , Tiedje, J.M. , and Cole, J.R. (2007) Naïve bayesian classifier for rapid assignment of rRNA sequences into the new bacterial taxonomy. Appl Environ Microbiol 73: 5261–5267. 17586664 10.1128/AEM.00062-07 PMC1950982

[mbt212733-bib-0055] Wilson, D.B. (2009) Cellulases and biofuels. Curr Opin Biotechnol 20: 295–299. 19502046 10.1016/j.copbio.2009.05.007

[mbt212733-bib-0056] Xia, Y. , Zhang, T. , and Fang, H.H. (2012) Thermophilic anaerobic degradation of microcrystalline cellulose using mixed culture enriched from anaerobic digestion sludge. Proc Environ Sci 12 (Part A): 3–8.

[mbt212733-bib-0057] Yokoyama, H. , Wagner, I.D. , and Wiegel, J. (2010) *Caldicoprobacter oshimai gen. nov., sp*. nov., an anaerobic, xylanolytic, extremely thermophilic bacterium isolated from sheep faeces, and proposal of Caldicoprobacteraceae fam. nov. Int J Syst Evol Microbiol 60 (Pt 1): 67–71. 19648349 10.1099/ijs.0.011379-0

[mbt212733-bib-0058] Yuan, Y. , Chen, Q. , Zhou, S. , Zhuang, L. , and Hu, P. (2012) Improved electricity production from sewage sludge under alkaline conditions in an insert‐type air‐cathode microbial fuel cell. J Chem Technol Biotechnol 87: 80–86.

[mbt212733-bib-0059] Zambare, V. , Zambare, A. , Muthukumarappan, K. , and Christopher, L.P. (2011) Biochemical characterization of thermophilic lignocellulose degrading enzymes and their potential for biomass bioprocessing. Int J Energy Environ 2: 99–112.

[mbt212733-bib-0060] Zavarzina, D.G. , Sokolova, T.G. , Tourova, T.P. , Chernyh, N.A. , Kostrikina, N.A. , and Bonch‐Osmolovskaya, E.A. (2007) *Thermincola ferriacetica* sp. nov., a new anaerobic, thermophilic, facultatively chemolithoautotrophic bacterium capable of dissimilatory Fe(III) reduction. Extremophiles 11: 1–7. 16988758 10.1007/s00792-006-0004-7

