# Supplementary material for: Simultaneous fermentation of cellulose and current production with an enriched mixed culture of thermophilic bacteria in a microbial electrolysis cell
Source: Microb Biotechnol. 2017 May 29;11(1):63–73. doi: 10.1111/1751-7915.12733 (PMC5743814; doi:10.1111/1751-7915.12733)
Supplement: Supplementary file 1 — Fig. S1. (a–f) Representative fermentation profiles tracked over 11 days from six serum bottles. Fig. S2. Picture of a thermophilic H‐type microbial electrochemical cell in an incubator at 60 °C. Fig. S3. CLSM LIVE/DEAD analysis revealed a live biofilm layer (Lf shown in yellow) approximately 40‐60 μm thick. [file MBT2-11-63-s001.pdf]

Supporting Information:

Simultaneous fermentation of cellulose and current production with an enriched mixed culture of thermophilic bacteria in a microbial electrolysis cell

Bradley G. Lusk,[1,2] Alexandra Colin,[3] Prathap Parameswaran,[4] Bruce E. Rittmann,[1,5] and Cesar I. Torres[1,6]

1 Biodesign Swette Center for Environmental Biotechnology, Arizona State University, P.O. Box 875701, Tempe,Arizona 85287-5701, United States

2 #ScienceTheEarth, www.sciencetheearth.com, Mesa, AZ, 85201

3 Ecole Normale Superieure, 45, rue d'Ulm, 75230 Paris Cedex 05, France

4 Department of Civil Engineering, Kansas State University, 2123 Fiedler Hall, Manhattan, Kansas 66502, United States of America

5 School of Sustainable Engineering and the Built Environment, Arizona State University, Tempe, Arizona, United States

6 School for Engineering of Matter, Transport and Energy, Arizona State University, Tempe, Arizona, United States

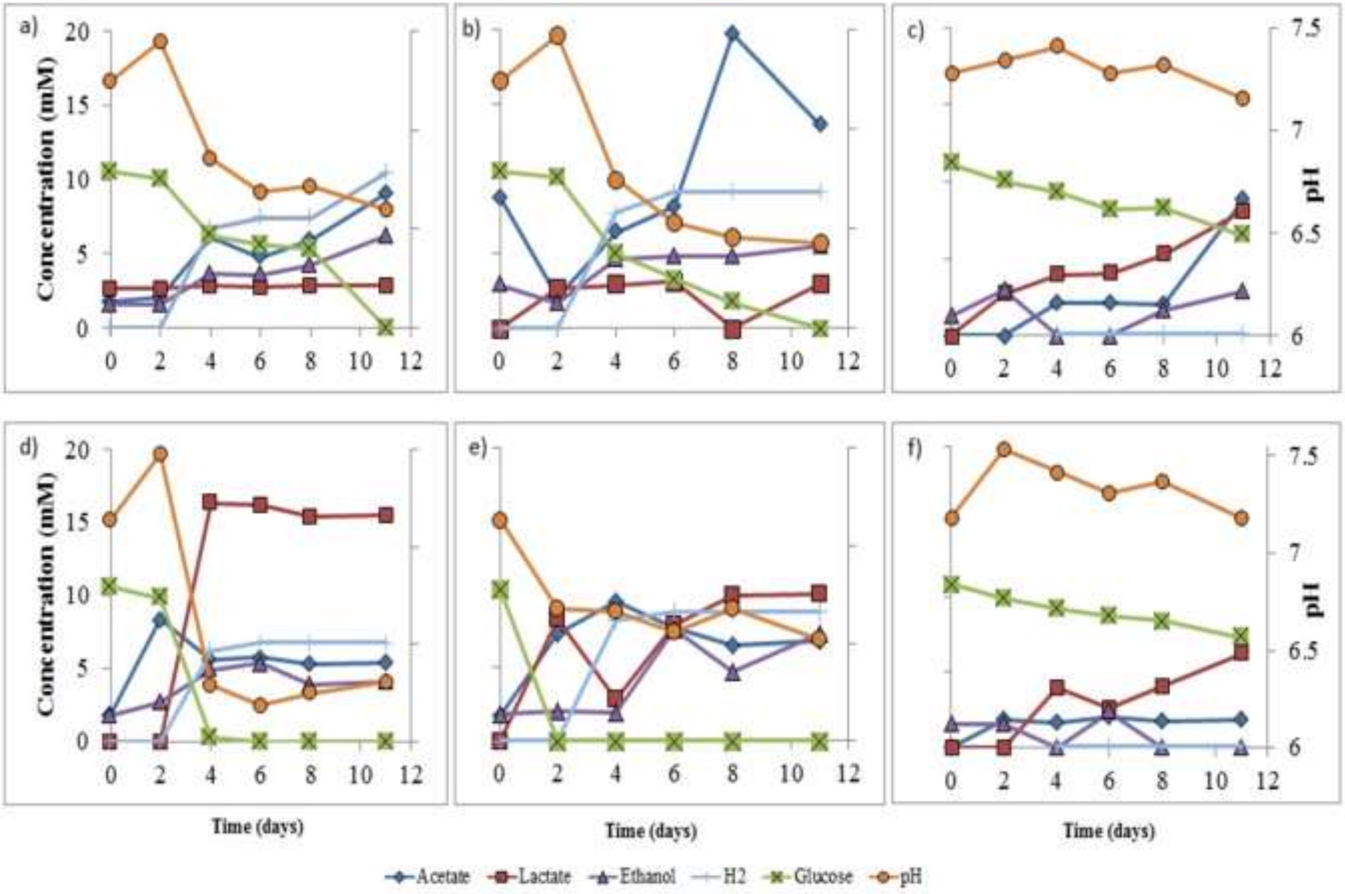

Figure S1a-f: Representative fermentation profiles tracked over 11 days from six serum bottles. The primary axis designates mM concentrations, while the secondary axis represents pH. Acetate is shown with (dark blue diamonds), lactate (red squares), ethanol (purple triangles), H_2 (light blue lines), and glucose (green squares with x's). Corresponding pH is indicated by orange circles. a-b show cellulolytic cultures grown with α-cellulose powder that were capable of cellulose fermentation while c shows a non-cellulolytic culture that was grown in the presence of α-cellulose power that was not capable of cellulose fermentation. d-e show cellulolytic cultures grown with cellulose filter paper that were capable of cellulose fermentation while f shows a non-cellulolytic culture that was grown in the presence of cellulose filter paper that was not capable of cellulose fermentation. Reactors d-e are representative samples of enriched celluloytic cultures that were used for MEC inoculation.

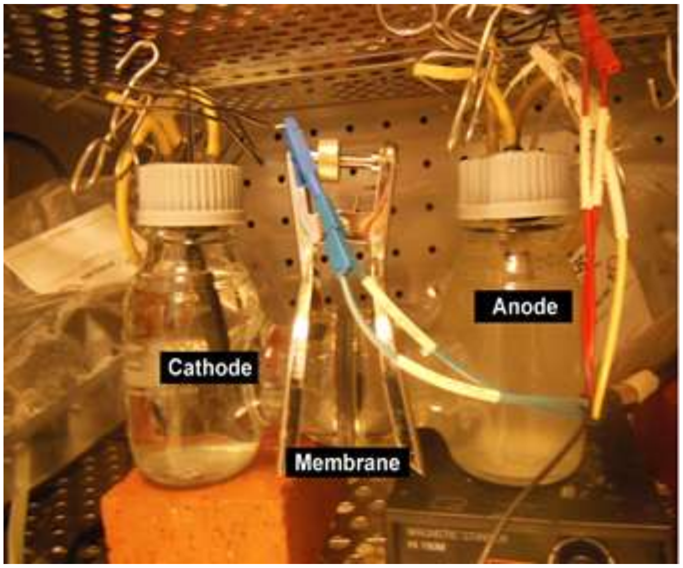

Figure S2: Picture of a thermophilic H-type microbial electrochemical cell in an incubator at 60ºC. On the left is a 350-mL cathode chamber, and on the right is a 350-mL anode chamber. The two chambers are separated by an anion exchange membrane. The anode (working electrode) is composed of two graphite electrodes, while the cathode (counter electrode) is composed of a single graphite electrode. The reference electrode is an Ag/AgCl reference electrode (BASi MF-2052). The blue wire is attached to the counter electrode, the red wire is attached to the working electrode, and the white wire is attached to the reference electrode. All wires are attached to a potentiostat (Princeton Applied Research, Model VMP3, Oak Ridge, TN). Gas collection bags are attached to the headspace of the anode and the cathode to capture volatile products in the gas phase.

Confocal Laser Scanning Microscopy LIVE/DEAD analysis (Figure S3) of the biofilms from MEC 1 revealed heterogeneous biofilm morphology with peaks and valleys. Live biofilm thickness (L_f) ranged between 40 to 60 µm. Similar L_f has been observed in thermophilic biofilms under similar operating conditions (Parameswaran 2013, Lusk 2016). At ~4 mM bicarbonate buffer and ~7 mM phosphate buffer, an L_f of 40 to 60 µm is sufficient to cause a proton gradient within the biofilm that may limit current production within the MEC (Torres 2008, Marcus 2011, Lusk 2016).

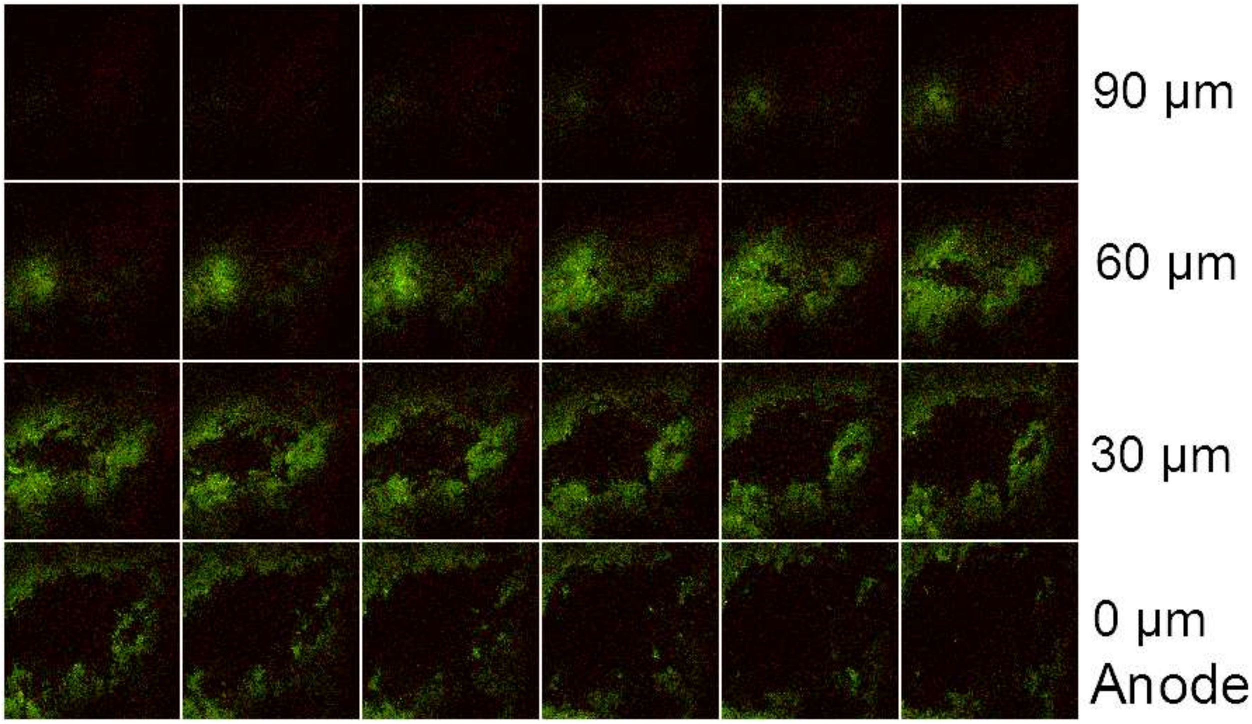

Figure S3: CLSM LIVE/DEAD analysis revealed a live biofilm layer (L_f shown in yellow) approximately 40-60 µm thick.

Modified DSMZ Medium 962: *Thermovenabulum* medium:

The medium consisted of the following in 1.0 L deionized water: 0.33 g each of NH_4Cl, KH_2PO_4, $MgCl_2*6H_2O$ and KCl; 0.1 g $CaCl_2*2H_2O$; 0.05 g yeast extract; 1 mL selenite-tungstate solution (prepared by dissolving 3 mg $Na_2SeO_3*5H_2O$, 4 mg $Na_2WO_4*2H_2O$ and 0.5 g NaOH in 1.0 L distilled water); 0.84 g $NaHCO_3$ (10 mM); 3.4 g of $NaCH_3COO*3H_2O$ (25 mM); 10 mM $Fe(OH)_3$ as electron acceptor; 10 mL ATCC vitamin solution; and 10 mL trace element solution. The trace elements solution consisted of the following ingredients in 1.0 L deionized water: 1.5 g nitrilotri-acetic acid, 3.0 g $MgSO_4*7H_2O$, 0.5 g $MnSO_4*H_2O$, 1.0 g NaCl, 0.1 g $FeSO_4*7H_2O$, 0.18 g $CoSO_4*7H_2O$, 0.1 g $CaCl_2*2H_2O$, 0.18 g $ZnSO_4*7H_2O$, 0.01 g $CuSO_4*5H_2O$, 0.02 g $KAl(SO_4)_2*12H_2O$, 0.01 g H_3BO_3, 0.01 g $Na_2MoO_4*2H_2O$, 0.03 g $NiCl_2*6H_2O$, and 0.3 mg $Na_2SeO_3*5H_2O$.

ATCC Medium 1190:

The medium consisted of the following in 1.0 L deionized water: 1.36 g KH_2PO_4 (10 mM); 4.2 g $Na_2HPO_4.12H_2O$ (2.5 mM); 0.5 g NH_4Cl; 0.18 g $MgCl_2.6H_2O$; 0.5 g yeast extract; 2.0 g glucose; 10 mL ATCC vitamin solution; 5 mL Wolfe's Modified Elixir; 40 mL Reducing Solution (prepared by dissolving 1 g $Na_2S.H_2O$ and 2.5 g l^{-1} Cysteine-HCl in 200 mL 0.2 N NaOH). Cellulose was provided by adding either Whatman #1 filter paper (Qualitative Circles, Cat no. 1001 042) with a diameter of 42.5 mm and an average weight of 0.120g at ~2.4g L^{-1} or α-Cellulose powder (Sigma) at 2.4g L^{-1}.

Quality control of the *T. ferriacetica*

Quality control of the *T. ferriacetica* culture was monitored using scanning electron microscopy coupled with 16S rDNA analysis. For this, we assembled a clone library following the protocol from a TOPO TA cloning kit with TOP10 *E. coli*. For acquiring sequences, DNA extracts from colonies were amplified using primers M13R and M13F. BLAST analysis revealed a 98-99% sequence similarity for all clones.
